# Concise textbook of child and adolescent psychiatry

**Published:** 2009

**Authors:** Vineet Kumar, Sandeep Grover

**Affiliations:** Department of Psychiatry, Postgraduate Institute of Medical Education and Research, Chandigarh - 160 012, India


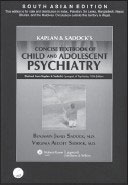


Child psychiatry is a growing subspecialty. The demand for evaluation and management of children and adolescents who may have mental disorders is increasing. More and more parents are now seeking advice to get the best out of their children who have behavioral, emotional, and cognitive difficulties or are apparently normal. Further, due to increasing awareness, atleast in big cities, mental disorders are now increasingly being recognized early. Due to availability of safer and efficacious pharmacologic agents, more and more patients are now being treated at younger ages. But there is a dearth of child psychiatrists to cater to this increasing demand in India. Therefore, every psychiatrist needs to have sound knowledge in child and adolescent psychiatry in today's world.

Kaplan and Sadock's *Concise Textbook of Child and Adolescent Psychiatry* (South Asian Edition) provides a comprehensive coverage of diagnosis and treatment of mental disorders in children and adolescents which have been listed in American Psychiatric Association's Diagnostic and Statistical Manual of Mental Disorders (DSM-IV-TR). This book has evolved from the author's experience editing Kaplan and Sadock's Synopsis of Psychiatry, tenth edition. This book has made a successful attempt to provide the latest information on diagnostic and management aspect of mental disorders in children and adolescents.

The book covers various areas in child psychiatry in 20 chapters, with one chapter specifically focusing on the treatment issues and another focusing on the forensic issues in child psychiatry. First chapter on clinical assessment which has focused on the way in which structured or semi-structured techniques (with respect to interviews, questionnaires and psychometrics) can be applied in the clinical context. Although, the description is brief and comprehensive, more space could have been give to techniques with respect to clinical interviews. The chapter on mental retardation covers the etiology comprehensively, besides the areas of assessment and management. This chapter also covers the concept of behavioural phenotypes, which are now increasingly recognized and are considered to provide better understanding about the biology of various developmental disorders in children in times to come. The chapters on learning disorders, motor skill disorders and communication disorders provide comprehensive information on the topics.

The chapter on pervasive developmental disorders gives a lucid description of pathophysiological understanding of role of genetic, immunologic, inflammatory and biochemical factors and dysfunctional neuronal connectivity besides the description of epidemiology, diagnosis, pathophysiology, differential diagnosis, course, prognosis and available treatment. With regard to treatment of autistic spectrum disorder the chapter highlights the role of behavioral and educational programs and also covers the evidence for use of various psychopharmacologic agents (escitalopram, venlafaxine, antipsychotics and tetrahydrobiopterin).

In the chapter on attention deficit disorder the authors have covered the various psychopharmacological agents comprehensively, including the monitoring issues, which are often neglected. However, drugs like clonidine, which is used quite frequently in a country like ours are not covered. The psychosocial intervention although has been described, it required more information about the practical techniques and tips.

The chapter on mood disorders highlights the fact that all antidepressants, as used in adults, have not been approved for children and adolescents and the clinicians should be aware that fluoxetine is the only antidepressant which has been approved by the FDA. The authors also discuss the issue of "black box" warning about use of antidepressants in children and adolescents and how the beneficial effects of antidepressants overshadow the suicidal behaviour associated with antidepressants use.

One of the most controversial issues in child psychiatry has been the diagnostic issues in early-onset bipolar disorders and it has been touched well. Another important issue i.e. the relationship between childhood bipolar disorder and ADHD has been mentioned briefly, but it would have been better if this issue could have been given more importance.

The chapter on anxiety disorders, provides a comprehensive description, however, the authors have mentioned about PANDAS (pediatric autoimmune neuropsychiatric disorders associated with Streptococcus) in passing. It would have been better to provide more information about the same considering the fact that streptococcal infections are quite common in developing countries setting.

There has recently been a significant increase in research dedicated to psychopharmacology in pediatric populations. The chapter on biological therapies cites the important findings of the recent trials pertaining to pediatric psychopharmacology i.e. the pediatric OCD treatment study (POTS), the treatment for adolescents with the depression study (TADS), the preschooler with ADHD treatment study (PATS) and the treatment of early onset schizophrenia spectrum disorders (TEOSS). This chapter has given a good overview of drug treatments of different childhood mental disorders.

The chapter on additional conditions that are at times the focus of clinical attention covers areas like issues of academic problems, childhood and adolescent antisocial behaviour and identity problem.

Finally the section on special areas of interest has added to the completeness of this book by describing some important issues like child custody, juvenile offenders, adverse life events and psychiatric symptoms, adoption and foster care, and child abuse. The description of the impact of terrorism on children in the form of psychologic disorders like acute stress disorder, PTSD, depression, anxiety, somatization, substance use etc is completely relevant considering the increasing frequency of terrorist attacks on the civilian population.

